# Primary, allied health, selected specialists, and mental health service utilisation by home care recipients in Australia before and after accessing the care, 2017–2019

**DOI:** 10.1007/s40520-024-02731-9

**Published:** 2024-03-29

**Authors:** Kailash Thapaliya, Gillian E. Caughey, Maria Crotty, Helena Williams, Steve L. Wesselingh, David Roder, Victoria Cornell, Gillian Harvey, Janet K. Sluggett, Tiffany K. Gill, Monica Cations, Jyoti Khadka, Andrew Kellie, Maria C. Inacio

**Affiliations:** 1 Registry of Senior Australians (ROSA), South Australian Health and Medical Research Institute, Adelaide, SA Australia; 2 UniSA Allied Health and Human Performance, University of South Australia, Adelaide, SA Australia; 3 College of Medicine and Public Health, Flinders University, Adelaide, SA Australia; 4 Southern Adelaide Local Health Network, SA Health, Adelaide, SA Australia; 5 Silverchain Group, Osborne Park, SA Australia; 6 South Australian Health and Medical Research Institute, Adelaide, SA Australia; 7 School of Public Health, The University of Adelaide, Adelaide, SA Australia; 8 Health and Social Care Economics Group, College of Nursing and Health Sciences, Flinders University, Bedford Park, SA Australia; 9 Adelaide Medical School, University of Adelaide, Adelaide, SA Australia; 10 College of Education, Psychology and Social Work, Flinders University, Bedford Park, SA Australia; 11 East Adelaide Healthcare, Newton, SA Australia

**Keywords:** Home care package, Primary care, General practitioner attendances, After-hour services, Allied health

## Abstract

**Objectives:**

To examine changes in primary, allied health, selected specialists, and mental health service utilisation by older people in the year before and after accessing home care package (HCP) services.

**Methods:**

A retrospective cohort study using the Registry of Senior Australians Historical National Cohort (≥ 65 years old), including individuals accessing HCP services between 2017 and 2019 (*N* = 109,558), was conducted. The utilisation of general practice (GP) attendances, health assessments, chronic disease management plans, allied health services, geriatric, pain, palliative, and mental health services, subsidised by the Australian Government Medicare Benefits Schedule, was assessed in the 12 months before and after HCP access, stratified by HCP level (1–2 vs. 3–4, i.e., lower vs. higher care needs). Relative changes in service utilisation 12 months before and after HCP access were estimated using adjusted risk ratios (aRR) from Generalised Estimating Equation Poisson models.

**Results:**

Utilisation of health assessments (7-10.2%), chronic disease management plans (19.7–28.2%), and geriatric, pain, palliative, and mental health services (all ≤ 2.5%) remained low, before and after HCP access. Compared to 12 months prior to HCP access, 12 months after, GP after-hours attendances increased (HCP 1–2 from 6.95 to 7.5%, aRR = 1.07, 95% CI 1.03–1.11; HCP 3–4 from 7.76 to 9.32%, aRR = 1.20, 95%CI 1.13–1.28) and allied health services decreased (HCP 1–2 from 34.8 to 30.7%, aRR = 0.88, 95%CI 0.87–0.90; HCP levels 3–4 from 30.5 to 24.3%, aRR = 0.80, 95%CI 0.77–0.82).

**Conclusions:**

Most MBS subsidised preventive, management and specialist services are underutilised by older people, both before and after HCP access and small changes are observed after they access HCP.

**Supplementary Information:**

The online version contains supplementary material available at 10.1007/s40520-024-02731-9.

## Introduction

Globally, the demographic shift towards an ageing population is evident, with the number of individuals aged 60 years and above steadily rising [1]. The United States, for example, has witnessed a growth rate in its population aged 65 years and above almost five times higher than the overall population between 1930 and 2020, with 55.8 million individuals (16.8% of its population) in this age group [2]. Likewise, the Australian population is ageing, with 4.2 million (16% of its population) aged 65 years and above in 2020 [3] and projected to increase to 21–23% by 2066 [3]. This demographic shift poses challenges in how the health and care needs of this population are met [4, 5]. As older individuals increasingly opt to age in place, global demand for services that enable them to live independently as long as possible has been observed [6, 7]. However, an older individual’s ability to live at home independently is significantly influenced by their health status [8].

In Australia, like other countries, demand and supply of services that facilitate older people to stay at home instead of being institutionalised have increased [9, 10]. While the programs have evolved, since 2016 there have been two home-based aged care service programs available, the Commonwealth Home Support Program (CHSP) and Home Care Packages (HCP) [11]. The CHSP offers one-off services for individuals, while HCPs offer bundled personal and clinical care services, with funding increasing with care needs [12]. The current HCP program provides four care levels, ranging from basic (level 1) to high-level (level 4) care needs [13].

The number of people accessing HCPs annually in Australia has increased 4.7-fold between 2011 and 2022, from 50,871 [14] to 236,928 [10]. During this period, those accessing HCP services have become frailer and have more health conditions and take more medications when starting care [15]. Additionally, the quality of care provided to these individuals has varied nationally and at times has been suboptimal [16]. For example, older people accessing HCPs are more likely to be hospitalised, have more emergency department (ED) presentations, and are less likely to access services like medication reviews, than individuals in residential aged care settings [17–19]. As more people wish and are remaining at home [20, 21], especially with documented increasing complex health profiles [22], understanding how their health care needs are managed is essential to identify and address potentially unmet needs [7, 23]. To understand this knowledge gap, this study examined access to and changes in primary, allied health, selected specialists, and mental health care service utilisation by new HCP service recipients.

## Method

### Study design, setting, and data sources

This population-based retrospective cohort study was conducted using the Registry of Senior Australians (ROSA) National Historical Cohort. As previously described, ROSA is a national platform that contains information on individuals who have accessed aged care services [24]. Briefly, ROSA contains integrated aged care, healthcare, and social welfare data. In this study, datasets within ROSA from the Australian Institute of Health and Welfare (AIHW) National Aged Care Data Clearinghouse (NACDC), which includes the National Death Index (NDI), and the Australian Government Medicare Benefits Schedule (MBS) and Pharmaceutical Benefits Scheme (PBS) were utilised. Linkage of these data sources are done at the individual level by the AIHW, which is a government accredited Integrating Authority.

### Study cohort

The study cohort included non-Aboriginal or Torres Strait Islander individuals aged ≥65 years, who did not have Department of Veterans’ Affairs (DVA) entitlements and received HCPs for the first time between 01/01/2017 and 31/12/2019 (Supplementary Fig. 1). The study cohort included all HCP levels and the stratified analysis was done by Levels 1–2 and Levels 3–4. The four HCP levels are: Level 1, basic care needs; Level 2, low-level care needs; Level 3, intermediate care needs; and Level 4, high-level care needs. These levels are recommended by clinically trained assessors after an aged care eligibility assessment, but HCP level accessed may also reflect HCP availability [13].

## Study outcomes

The utilisation of Australian Government MBS subsidised healthcare services in the 12 months before and after HCP access were the outcomes of interest. Australia has an universal health care system, which subsidises medical services (including general practitioner (GP), allied health and some specialist attendances), hospital, and medications provided to residents [25, 26]. The MBS subsidised health care services examined were categorised into: (1) general attendances with GPs, medical or nurse practitioners; (2) GP health assessments and management plans; (3) allied health services (i.e., optometrical services, comprehensive medication reviews, dentistry, and allied health services part of chronic disease management plan); (4) mental health services; and (5) selected specialist attendances, which included pain, palliative, and geriatric medicine attendances. See Supplementary Table 1 for all MBS services included and their items.

### Covariates

Covariates included age, sex, state/territory where HCP service was obtained, remoteness (major cities, inner regional, outer regional/remote/very remote), number of health conditions (categorised as 0–4, 5–6, and 7 and more), dementia status, and service provider type (government, not-for-profit, and private). Number of health conditions were ascertained using the Rx-Risk-V pharmaceutical based comorbidity index [27] in the 6 months prior to study entry. Dementia was ascertained using the Rx-Risk-V and aged care eligibility assessment [28].

### Statistical analysis

All analyses were conducted by HCP levels (Levels 1–2 and Levels 3–4). Characteristics of the study cohort were summarised using descriptive statistics, including frequencies and percentages for categorical variables, and medians and interquartile ranges (IQRs) for continuous variables. Services utilisation was examined 12 months before and after HCP service initiation (time 0) by 3 months intervals. The denominator for each 3 months interval included individuals who were alive within the interval and met the service eligibility criteria (e.g., time and age). For example, for the utilisation of health assessments, only individuals aged 75 + years without an assessment in the prior 12 months were included in the denominator [29]. Adjusted (age, sex, comorbidities, and state) health service utilisation is shown as incidence proportions and 95% confidence intervals (CI). The adjusted relative changes in service 12 months after HCP access compared to 12 months before were estimated using adjusted risk ratios (aRR) calculated using generalised estimating equation Poisson models. There was minimal missing data (*n* = 560/109,558 [0.5%] of individuals had no MBS records, and *n* = 34/109,558 [< 0.1%] had missing covariate information) and a complete case analysis was performed.

Individuals who accessed the HCP services in the two least populous states (i.e., Australian Capital Territory (ACT, *N* = 1497) and Northern Territory (NT, *N* = 245)) were excluded from selected analyses due to low service utilisation (as noted in tables). All analyses were conducted using SAS 9.4 (SAS Institute, Cary, NC, USA).

## Results

### Demography

Of the 109,558 individuals studied (Supplementary Fig. 1), 74.2% (*n* = 81,500) accessed HCP levels 1–2, 61.2% (*n* = 67,123) were female, and 21.2% (*n* = 23,313) had a diagnosis of dementia. The median age was 82 years (IQR 77–87) and the median comorbidity score was 5 (IQR 3–7). Within a year of HCP entry, 10.0% (*n* = 11,026) of the HCP recipients died and 15.4% (*n* = 16,942) of the recipients transitioned to permanent residential aged care (Table 1).

**Table 1:** Study cohort characteristics, 2017–2019

Variables	Total (*n* = 109,558)	HCP levels 1–2 (*n* = 81,500)	HCP levels 3–4 (*n* = 28,058)
*Age, years Median (IQR)*	82 (77–87)	83 (77–87)	82 (76–87)
*Age category*			
65–74	20,051 (18.3)	13,914 (17.0)	6137 (21.9)
75–84	46,856 (42.7)	35,494 (43.6)	11,362 (40.5)
85–94	39,951 (36.4)	30,315 (37.2)	9636 (34.3)
95 and above	2700 (2.5)	1777 (2.2)	923 (3.3)
*Sex*			
Female	67,123 (61.2)	50,718 (62.2)	16,405 (58.5)
Male	42,434 (38.7)	30,781 (37.8)	11,653 (41.5)
Missing	1 (< 0.01)	-	-
*Service state*			
New South Wales	38,117 (34.7)	29,529 (36.2)	8588 (30.6)
Victoria	25,468 (23.2)	19,285 (23.7)	6183 (22.1)
Queensland	23,711 (21.6)	16,949 (20.8)	6762 (24.1)
Western Australia	9772 (8.92)	6311 (7.70)	3461 (12.3)
South Australia	8238 (7.50)	6315 (7.70)	1923 (6.80)
Tasmania	2518 (2.30)	1873 (2.30)	645 (2.30)
Australian Capital Territory	1490 (1.36)	1083 (1.30)	407 (1.40)
Northern Territory	244 (0.22)	155 (0.20)	89 (0.30)
*Remoteness*			
Major cities	75,250 (68.6)	55,572 (68.2)	19,678 (70.1)
Inner regional	26,945 (24.5)	20,638 (25.3)	6307 (22.4)
Outer regional/remote/very remote	7330 (6.69)	5265 (6.46)	2065 (7.37)
Missing	33 (0.02)	25 (0.03)	8 (0.03)
*Count of comorbid conditions-Rx-Risk* ^*a*^			
0–4	43,487 (39.6)	32,475 (39.8)	11,012 (39.3)
5–6	31,832 (29.0)	23,976 (29.4)	7856 (28.0)
7+	34,239 (31.2)	25,049 (30.8)	9190 (32.7)
Median (IQR)	5 (3–7)	5 (3–7)	5 (3–7)
*Dementia diagnosis* ^*b*^	23,313 (21.2)	15,054 (18.4)	8259 (29.4)
*Type of service provider*			
Government	7115 (6.48)	5469 (6.71)	1646 (5.80)
Not-for-profit	75,349 (68.7)	55,889 (68.6)	19,460 (69.3)
Private	27,094 (24.7)	20,142 (24.7)	6952 (24.9)

^a^Determined using Rx-Risk-V pharmaceutical based comorbidity index [27]

^b^Dementia ascertained using Rx-Risk-V and aged care eligibility assessment [28]

*Abbreviations* IQR, Interquartile range; HCP, Home Care Package

### Overall service utilisation

In the overall cohort, GP attendances utilisation was high (ranging from 79.5 to 91%) and the utilisation of health assessments (ranging from 7 to 10.2%), chronic disease management plans (ranging from 19.7 to 28.2%), geriatric attendances (ranging from 1 to 2%), pain specialist attendances (< 1%), palliative specialists attendances (< 1%), and mental health services (< 2.5%) remained low both before and after accessing HCP care (Supplementary Table 2).

### Service utilisation 12 months before and after accessing HCP

**Fig. 1:**
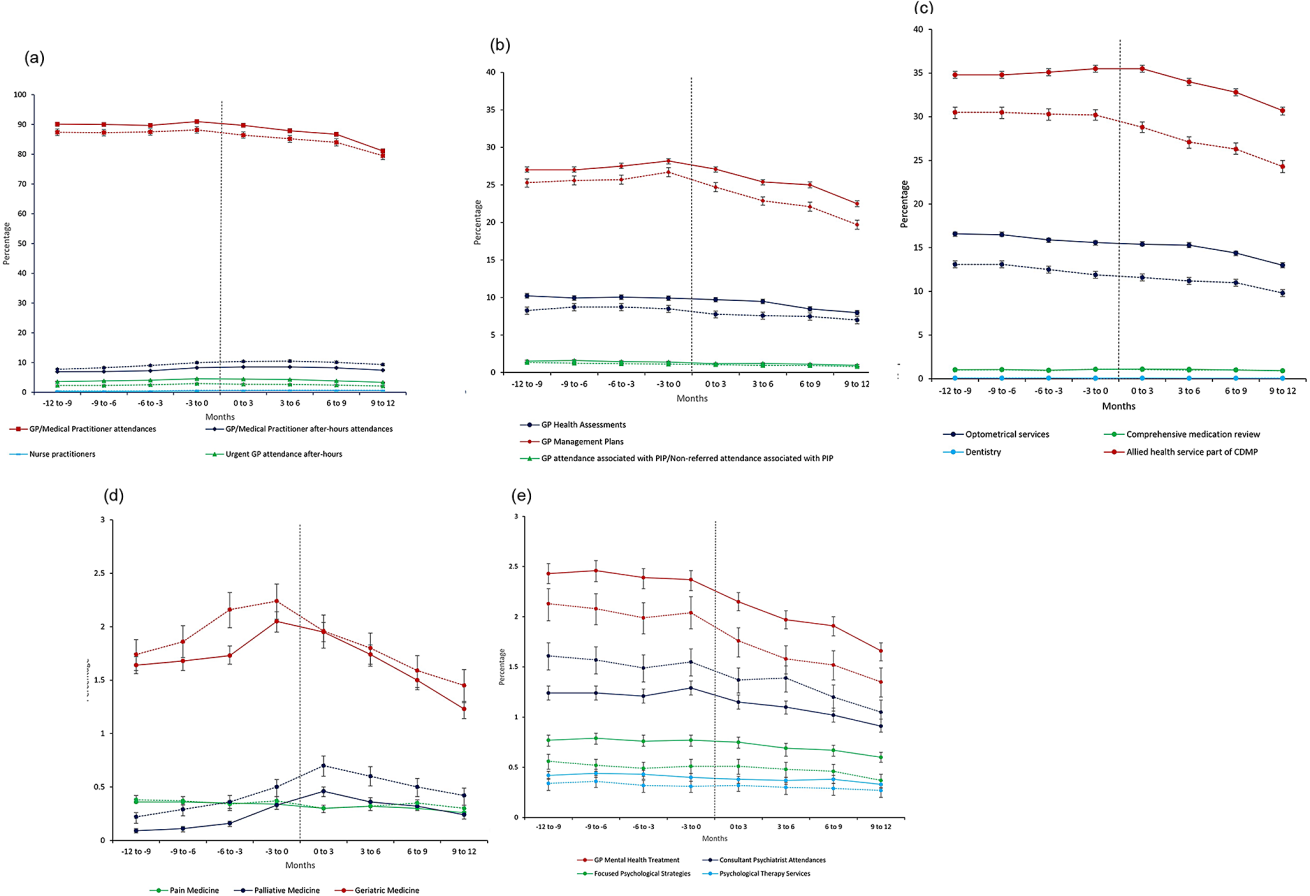
Adjusted incidence proportions of Medicare Benefits Schedule subsidised primary care (general attendances [**A**], health assessments/management plans[**B**]), allied health (**C**), pain, palliative, and geriatric (**D**) and mental health (**E**) services among individuals accessing Home Care Packages (HCP), 2017-2019. *Note.* Continuous lines indicate HCP levels 1-2 and dotted lines indicate HCP levels 3-4 Abbreviations GP, General Practitioner; PIP, Practice Incentives Program

Compared to the 12 months prior to HCP access, in the 12 months after, there was a 10% decrease (aRR 0.90, 95%CI 0.89–0.91) in GP or medical practitioner general attendances by HCP recipients receiving HCP levels 1–2, from 90.1% (95%CI 89.5–90.8) to 81.1% (95% CI 80.4–81.8), and an 9% decrease (aRR 0.91, 95%CI 0.89–0.93) by HCP recipients receiving HCP levels 3–4, from 87.4% (95%CI 86.3–88.5) to 79.5% (95%CI 78.3–80.8) (Tables 2, 3 and 4; Fig. 1). Utilisation of GP or medical practitioner after-hours services increased by 7% (aRR 1.07, 95%CI 1.03–1.11) for HCP levels 1–2, from 6.95% (95%CI 6.77–7.13) to 7.45% (95%CI 7.24–7.65) and increased by 20% (aRR 1.20, 95%CI 1.13–1.28) for HCP levels 3–4, from 7.76% (95%CI 7.45–8.72) to 9.32% (95%CI 8.92–9.74), with 10% (aRR 0.90, 95%CI 0.84–0.96) decreases in urgent after-hours GP or medical practitioner attendances for HCP level 1–2, from 2.33% (95%CI 2.2–2.4) to 2.11% (95%CI 2.00-2.22). GP health assessments decreased by 22% (aRR 0.78, 95%CI 0.74–0.82) for HCP levels 1–2, from 10.2% (95%CI 9.91–10.5) to 7.98% (95%CI 7.69–8.29), and decreased by 15% (aRR 0.85, 95%CI 0.77–0.93) for HCP levels 3–4, from 8.26% (95%CI 7.80–8.76) to 7.00% (95%CI 6.52–7.52). GP management plans decreased by 17% (aRR 0.83, 95%CI 0.82–0.85) for HCP levels 1–2, from 27.0% (95%CI 26.6–27.3) to 22.5% (95%CI 22.1–22.9), and 22% (aRR 0.78, 95%CI 0.75–0.81) for HCP levels 3–4, from 25.3% (95% CI 24.8–25.9) to 19.7% (95%CI 19.1–20.3) (Tables 2, 3 and 4; Fig. 1).

**Table 2:** Adjusted^a^ incidence proportion of Medicare Benefits Schedule subsidised primary care, allied health, mental health, geriatric, pain and palliative services among Home Care Package (HCP) recipients by 3-month interval in the 12 months before their HCP access

Service Group / HCP Level	-12 to -9 months *N* = 109,558		-9 to -6 months *N* = 109,558		-6 to -3 months *N* = 109,558		-3 to 0 months *N* = 109,558	
	HCP L 1–2 *n* = 81,500	HCP L 3–4 *n* = 28,058	HCP L 1–2 *n* = 81,500	HCP L 3–4 *n* = 28,058	HCP L 1–2 *n* = 81,500	HCP L 3–4 *n* = 28,058	HCP L 1–2 *n* = 81,500	HCP L 3–4 *n* = 28,342
Primary Care	Adjusted IP (95%CI)	Adjusted IP (95%CI)	Adjusted IP (95%CI)	Adjusted IP (95%CI)	Adjusted IP (95%CI)	Adjusted IP (95%CI)	Adjusted IP (95%CI)	Adjusted IP (95%CI)
*General Attendances*								
GP/Medical practitioner attendances	90.1 (89.5–90.8)	87.4 (86.3–88.5)	90.0 (89.4–90.6)	87.3 (86.3–88.4)	89.7 (89.1–90.4)	87.5 (86.4–88.6)	91.0 (91.4–91.7)	88.2 (87.1–89.3)
Urgent GP attendance after-hours	2.33 (2.2–2.4)	3.61 (3.39–3.83)	2.30 (2.20–2.41)	3.87 (3.65–4.11)	2.52 (2.42–2.63)	4.08 (3.85–4.32)	2.94 (2.83–3.06)	4.56 (4.32–4.81)
GP/Medical practitioner after-hours attendances	6.95 (6.77–7.13)	7.76 (7.45–8.72)	6.98 (6.81–7.17)	8.28 (7.95–8.62)	7.25 (7.07–7.45)	9.02 (8.68–9.38)	8.27 (8.07–8.46)	9.97 (9.61–10.3)
Nurse practitioners	0.36 (0.32–0.40)	0.50 (0.43–0.48)	0.36 (0.33–0.41)	0.48 (0.41–0.56)	0.40 (0.36–0.45)	0.48 (0.41–0.57)	0.46 (0.42–0.51)	0.68 (0.60–0.78)
*Health Assessments / Management Plans*								
GP Health assessments^b^	10.2 (9.91–10.53)	8.26 (7.80–8.76)	9.93 (9.63–10.24)	8.73 (8.26–9.24)	10.0 (9.74–10.36)	8.73 (8.26–9.23)	9.91 (9.62–10.22)	8.49 (8.03–8.97)
GP Management plans	27.0 (26.6–27.3)	25.3 (24.8–25.9)	27.0 (26.6–27.3)	25.6 (25.0-26.2)	27.5 (27.1–27.8)	25.7 (25.1–26.3)	28.2 (27.9–28.6)	26.7 (26.1–27.3)
GP Attendance associated with PIP/Non-referred attendance associated with PIP	1.52 (1.44–1.60)	1.34 (1.22–1.48)	1.59 (1.51–1.68)	1.25 (1.13–1.38)	1.46 (1.38–1.54)	1.17 (1.06–1.30)	1.39 (1.31–1.46)	1.12 (1.01–1.24)
*Allied Health Services*								
Optometrical services	16.6 (16.4–16.9)	13.1 (12.7–13.6)	16.5 (16.2–16.7)	13.1 (12.7–13.5)	15.9 (15.7–16.2)	12.5 (12.1–13.0)	15.6 (15.4–15.9)	11.9 (11.5–12.3)
Comprehensive medication review	1.04 (0.97–1.11)	1.00 (0.89–1.12)	1.05 (0.98–1.12)	1.02 (0.91–1.14)	0.99 (0.92–1.06)	0.94 (0.83–1.05)	1.07 (1.01–1.15)	1.10 (0.99–1.23)
Dentistry^c,d^	0.07 (0.06–0.09)	0.07 (0.04–0.10)	0.06 (0.05–0.08)	0.09 (0.06–0.13)	0.07 (0.06–0.09)	0.08 (0.05–0.11)	0.07 (0.06–0.09)	0.03 (0.02–0.06)
Allied health service part of CDMP	34.8 (34.4–35.2)	30.5 (29.9–31.2)	34.8 (34.4–35.2)	30.5 (29.9–31.2)	35.1 (34.7–35.5)	30.3 (29.7–31.0)	35.5 (35.1–35.9)	30.2 (29.6–30.8)
*Mental Health Services*								
GP Mental health treatment	2.43 (2.33–2.53)	2.13 (1.98–2.30)	2.46 (2.36–2.57)	2.08 (1.93–2.24)	2.39 (2.30–2.50)	1.99 (1.84–2.15)	2.37 (2.28–2.48)	2.04 (1.88–2.20)
Consultant psychiatrist attendances	1.24 (1.17–1.31)	1.61 (1.48–1.75)	1.24 (1.17–1.31)	1.57 (1.44–1.71)	1.21 (1.14–1.28)	1.49 (1.36–1.63)	1.29 (1.22–1.36)	1.55 (1.42–1.69)
Focused psychological strategies	0.77 (0.72–0.83)	0.56 (0.49–0.64)	0.79 (0.74–0.85)	0.52 (0.46–0.60)	0.76 (0.70–0.81)	0.49 (0.43–0.57)	0.77 (0.72–0.83)	0.51 (0.44–0.58)
Psychological therapy services^d^	0.42 (0.38–0.46)	0.34 (0.29–0.41)	0.44 (0.40–0.48)	0.36 (0.30–0.42)	0.43 (0.39–0.48)	0.32 (0.27–0.39)	0.40 (0.36–0.44)	0.31 (0.26–0.37)
*Specialist Geriatric, Pain and Palliative Services*								
Pain medicine^d^	0.36 (0.32–0.39)	0.38 (0.32–0.45)	0.36 (0.32–0.40)	0.37 (0.31–0.44)	0.35 (0.32–0.39)	0.34 (0.29–0.41)	0.34 (0.30–0.38)	0.37 (0.32–0.45)
Palliative medicine^d^	0.09 (0.07–0.11)	0.22 (0.18–0.28)	0.11 (0.09–0.14)	0.29 (0.24–0.35)	0.16 (0.14–0.19)	0.36 (0.30–0.44)	0.33 (0.30–0.37)	0.50 (0.43–0.59)
Geriatric medicine	1.64 (1.56–1.72)	1.74 (1.60–1.89)	1.68 (1.68–1.77)	1.86 (1.71–2.01)	1.73 (1.64–1.81)	2.16 (2.00-2.33)	2.05 (1.96–2.15)	2.24 (2.08–2.41)

^a^Adjusted for sex, age category, number of comorbidities and state of residence

^b^Only individuals 75 + years old were eligible and only once/year, therefore cohort restricted to 75 + only (-12 to -9 months, *n* = 53,157; -9 to -6 months, *n* = 53,953; -6 to -3 months, *n* = 54,691; -3 to 0 months, *n* = 55,412)

^c^Australian Capital Territory (*n* = 1497) excluded from the analyses due to low service utilization

^d^Northern Territory (*n* = 245) excluded from the analyses due to low service utilization

*Abbreviations* HCP, Home Care Package; L, Levels; GP, General Practitioner; PIP, Practice Incentive Program; IP, Incidence Proportion; CI, Confidence Interval; CDMP, Chronic Disease Management Plan

**Table 3:** Adjusted^a^ incidence proportion of Medicare Benefits Schedule subsidised primary care, allied health, mental health, geriatric, pain and palliative services among Home Care Package (HCP) recipients by 3-month interval in the 12 months after their HCP access

Service Group / HCP Level	0 to 3 months *N* = 109,558		3 to 6 months *N* = 102,264		6 to 9 months *N* = 94,032		9 to 12 months *N* = 87,140	
	HCP L 1–2 *n* = 81,500	HCP L 3–4 *n* = 28,058	HCP L 1–2 *n* = 76,649	HCP L 3–4 *n* = 25,615	HCP L 1–2 *n* = 71,017	HCP L 3–4 *n* = 23,015	HCP L 1–2 *n* = 66,282	HCP L 3–4 *n* = 20,858
*Primary Care*	**Adjusted IP** **(95%CI)**	**Adjusted IP** **(95%CI)**	**Adjusted IP** **(95%CI)**	**Adjusted IP** **(95%CI)**	**Adjusted IP** **(95%CI)**	**Adjusted IP** **(95%CI)**	**Adjusted IP** **(95%CI)**	**Adjusted IP** **(95%CI)**
*General Attendances*								
GP/Medical practitioner attendances	89.7 (89.0-90.3)	86.4 (85.3–87.4)	87.9 (87.2–88.6)	85.2 (84.1–86.4)	86.7 (86.0-87.4)	84.0 (82.8–85.2)	81.1 (80.4–81.8)	79.5 (78.3–80.8)
Urgent GP attendance after-hours	2.74 (2.63–2.85)	4.47 (4.23–4.72)	2.68 (2.57–2.80)	4.31 (4.07–4.57)	2.42 (2.31–2.54)	3.87 (3.63–4.13)	2.11 (2.00-2.22)	3.37 (3.13–3.62)
GP/Medical practitioner after-hours attendances	8.54 (8.35–8.74)	10.4 (10.1–10.8)	8.53 (8.33–8.74)	10.5 (10.1–10.9)	8.25 (8.04–8.46)	10.1 (9.65–10.5)	7.45 (7.24–7.65)	9.32 (8.92–9.74)
Nurse practitioners	0.55 (0.50–0.60)	0.70 (0.61–0.79)	0.56 (0.51–0.61)	0.74 (0.65–0.85)	0.54 (0.49–0.60)	0.71 (0.61–0.82)	0.53 (0.48–0.58)	0.67 (0.57–0.78)
*Health Assessments / Management Plans*								
GP Health assessments^b^	9.71 (9.42–10.01)	7.75 (7.31–8.22)	9.48 (9.18–9.79)	7.59 (7.14–8.08)	8.48 (8.19–8.79)	7.47 (6.99–7.97)	7.98 (7.69–8.29)	7.00 (6.52–7.52)
GP Management plans	27.1 (26.8–27.5)	24.7 (24.1–25.3)	25.4 (25.1–25.8)	22.9 (22.4–23.5)	25.0 (24.6–25.4)	22.1 (21.5–22.7)	22.5 (22.1–22.9)	19.7 (19.1–20.3)
GP Attendance associated with PIP/Non-referred attendance associated with PIP	1.18 (1.12–1.26)	1.05 (0.95–1.17)	1.20 (1.13–1.28)	0.94 (0.84–1.06)	1.10 (1.03–1.17)	0.93 (0.82–1.06)	0.96 (0.89–1.03)	0.80 (0.69–0.92)
*Allied Health Services*								
Optometrical services	15.4 (15.1–15.6)	11.6 (11.2–12.0)	15.3 (15.0-15.6)	11.2 (10.8–11.6)	14.4 (14.2–14.7)	11.0 (10.5–11.4)	13.0 (12.7–13.3)	9.81 (9.40–10.2)
Comprehensive medication review	1.12 (1.05–1.19)	1.05 (0.93–1.17)	1.08 (1.01–1.19)	0.98 (0.87–1.11)	1.00 (0.93–1.07)	1.02 (0.90–1.15)	0.93 (0.86-1.00)	0.89 (0.78–1.03)
Dentistry^c,d^	0.07 (0.05–0.09)	0.06 (0.04–0.10)	0.06 (0.04–0.08)	0.06 (0.03–0.09)	0.06 (0.05–0.08)	0.06 (0.04–0.10)	0.05 (0.04–0.07)	0.07 (0.05–0.12)
Allied health service part of CDMP	35.5 (35.1–35.9)	28.8 (28.2–29.4)	34.0 (33.6–34.4)	27.1 (26.5–27.8)	32.8 (32.4–33.2)	26.3 (25.6–26.9)	30.7 (30.3–31.2)	24.3 (23.6–25.0)
*Mental Health Services*								
GP Mental health treatment	2.15 (2.06–2.24)	1.76 (1.63–1.92)	1.97 (1.88–2.06)	1.58 (1.45–1.73)	1.91 (1.82–2.01)	1.52 (1.38–1.67)	1.66 (1.58–1.76)	1.35 (1.21–1.50)
Consultant psychiatrist attendances	1.15 (1.08–1.22)	1.37 (1.25–1.50)	1.10 (1.04–1.17)	1.39 (1.27–1.53)	1.02 (0.95–1.09)	1.20 (1.08–1.34)	0.91 (0.84–0.97)	1.05 (0.93–1.18)
Focused psychological strategies	0.75 (0.70–0.81)	0.51 (0.44–0.59)	0.69 (0.64–0.77)	0.48 (0.41–0.56)	0.67 (0.62–0.73)	0.46 (0.39–0.53)	0.60 (0.55–0.65)	0.37 (0.31–0.44)
Psychological therapy services^d^	0.38 (0.35–0.42)	0.32 (0.26–0.38)	0.37 (0.34–0.41)	0.30 (0.25–0.37)	0.38 (0.34–0.42)	0.29 (0.24–0.36)	0.33 (0.29–0.37)	0.27 (0.22–0.34)
*Specialist Geriatric, Pain and Palliative Services*								
Pain medicine^d^	0.30 (0.27–0.34)	0.30 (0.24–0.36)	0.32 (0.29–0.36)	0.32 (0.27–0.39)	0.30 (0.27–0.34)	0.35 (0.29–0.42)	0.26 (0.23–0.30)	0.30 (0.24–0.37)
Palliative medicine^d^	0.46 (0.42–0.51)	0.70 (0.61–0.80)	0.36 (0.32–0.40)	0.60 (0.51–0.69)	0.32 (0.29–0.36)	0.50 (0.42–0.59)	0.24 (0.21–0.28)	0.42 (0.35–0.51)
Geriatric medicine	1.95 (1.86–2.04)	1.96 (1.81–2.12)	1.74 (1.65–1.83)	1.80 (1.66–1.97)	1.50 (1.42–1.59)	1.59 (1.45–1.75)	1.23 (1.16–1.32)	1.45 (1.30–1.61)

^a^Adjusted for sex, age category, number of comorbidities and state of residence

^b^Only individuals 75 + years old were eligible and only once/year, therefore cohort restricted to 75 + only (0 to 3 months, *n* = 56,124; 3 to 6 months, *n* = 52,825; 6 to 9 months, *n* = 48,934; 9 to 12 months, *n* = 46,136)

^c^Australian Capital Territory (*n* = 1497) excluded from the analyses due to low service utilization

^d^Northern Territory (*n* = 245) excluded from the analyses due to low service utilization

*Abbreviations* HCP, Home Care Package; L, Levels; GP, General Practitioner; PIP, Practice Incentive Program; IP, Incidence Proportion; CI, Confidence Interval; CDMP, Chronic Disease Management Plan

**Table 4:** Adjusted^a^ risk ratio and 95% confidence interval of Medicare Benefits Schedule subsidised health care utilisation 12 months before and after Home Care Package access, 2017–2019

Service Group / HCP Level	12 months before and after HCP access	
Primary Care	Adjusted Risk Ratio (95% CI)^e^	
General Attendances	HCP Levels 1–2	HCP Levels 3–4
GP/Medical practitioner attendances	0.90 (0.89–0.91)	0.91 (0.89–0.93)
Urgent GP attendance after-hours	0.90 (0.84–0.96)	0.93 (0.85–1.03)
GP/Medical practitioner after-hours attendances	1.07 (1.03–1.11)	1.20 (1.13–1.28)
Nurse practitioners	1.47 (1.27–1.70)	1.34 (1.08–1.66)
*Health Assessments / Management Plans*		
GP Health assessments^b^	0.78 (0.74–0.82)	0.85 (0.77–0.93)
GP Management plans	0.83 (0.82–0.85)	0.78 (0.75–0.81)
GP attendance associated with PIP/Non-referred attendance associated with PIP	0.63 (0.58–0.69)	0.59 (0.50–0.70)
*Allied Health Services*		
Optometrical services	0.78 (0.76–0.80)	0.75 (0.71–0.79)
Comprehensive medication review	0.88 (0.80–0.98)	0.90 (0.76–1.08)
Dentistry^c,d^	0.71 (0.47–1.07)	1.13 (0.59–2.15)
Allied health service part of CDMP	0.88 (0.87–0.90)	0.80 (0.77–0.82)
*Mental Health Services*		
GP Mental health treatment	0.69 (0.64–0.73)	0.63 (0.56–0.72)
Consultant psychiatrist attendances	0.72 (0.66–0.79)	0.65 (0.57–0.75)
Focused psychological strategies	0.76 (0.69–0.85)	0.66 (0.53–0.81)
Psychological therapy services^d^	0.79 (0.68–0.91)	0.79 (0.61–1.02)
*Specialist Geriatric, Pain and Palliative Services*		
Pain medicine^d^	0.74 (0.63–0.87)	0.79 (0.60–1.03)
Palliative medicine^d^	2.67 (2.08–3.44)	1.88 (1.41–2.52)
Geriatric medicine	0.76 (0.70–0.82)	0.83 (0.73–0.95)

^a^Adjusted for sex, age category, number of comorbidities and state of residence

^b^Only individuals 75 + years old were eligible and only once/year, therefore cohort restricted to 75 + only (-12 to -9 months, *n* = 53,157; -9 to -6 months, *n* = 53,953; -6 to -3 months, *n* = 54,691; -3 to 0 months, *n* = 55,412; 0 to 3 months, *n* = 56,124; 3 to 6 months, *n* = 52,825; 6 to 9 months, *n* = 48,934; 9 to 12 months, *n* = 46,136)

^c^Australian Capital Territory (*n* = 1497) excluded from the analyses due to low service utilization

^d^Northern Territory (*n* = 245) excluded from the analyses due to low service utilization

^e^Reference category = 12 months before HCP

*Abbreviations* MBS, Medicare Benefits Schedule; HCP, Home Care Package; GP, General Practitioner; PIP, Practice Incentive Program; CI, Confidence Interval; CDMP, Chronic Disease Management Plan

The utilisation of allied health services offered as part of the chronic disease management plans decreased by 12% (aRR 0.88, 95%CI 0.87–0.90) for HCP levels 1–2, from 34.8% (95%CI 34.4–35.2) to 30.7% (95%CI 30.3–31.2), and decreased by 20% (aRR 0.80, 95%CI 0.77–0.82) for HCP levels 3–4, from 30.5% (95%CI 29.9–31.2) to 24.3% (95%CI 23.6–25.0). Podiatry services accounted for 83% of these allied health services (Supplementary Table 2). Utilisation of comprehensive medication review decreased by 12% (aRR 0.88, 95%CI 0.80–0.98) for HCP levels 1–2, from 1.04% (95%CI 0.97–1.11) to 0.93% (95%CI 0.83-1.00), with little change for HCP levels 3–4 (Tables 2, 3 and 4; Fig. 1).

The utilisation of GP mental health treatment services decreased by 31% (aRR 0.69 95%CI 0.64–0.73) for HCP levels 1–2, from 2.43% (95%CI 2.33–2.53) to 1.66% (95%CI 1.58–1.76), and 37% (aRR 0.63, 95%CI 0.56–0.72) for HCP levels 3–4, from 2.13% (95%CI 1.98–2.30) to 1.35% (95%CI 1.21–1.50). While use of specialist palliative services was low (0.1–0.4%) over the study period, it increased 2.67 fold (aRR 2.67, 95%CI 2.08–3.44) after HCP access, and almost 2-fold (aRR 1.88, 95%CI 1.41–2.52) for HCP levels 3–4. Access to geriatric medicine was also low (1.23–2.24%) over the time period, and decreased 24% (aRR = 0.76, 95%CI 0.70–0.82) in HCP levels 1–2 and 17% (aRR = 0.83, 95%CI 0.73–0.95) for HCP level 3–4 (Tables 2, 3 and 4; Fig. 1).

## Discussion

This national investigation into government subsidised health care service utilisation of older Australians receiving HCP services has determined that except for GP attendances, all other services examined, including health assessment, management plans, allied health services, pain, palliative, geriatric, and mental health services, were underutilised by people before and after accessing HCPs. We also determined that small changes, mostly decreases, in utilisation of these services were observed after individuals accessed HCP services.

The observed low and decreasing trend of utilisation of MBS subsidised services among older people living at home, who have sought and obtained HCP services because of their inability to continue to live at home unassisted [13, 30], highlights potential areas in need of improvement regarding health care access. People accessing HCPs have a well-documented high burden of multimorbidity (median comorbidity score = 5), frequently experience polypharmacy (median medications = 9), often experience a high sedative load in a year (29.1%), a high proportion are chronic opioid users (13.7%), have a high prevalence of mental health conditions (e.g., depression 36%), and are frequently hospitalised (43% go to the ED at least one time a year, 11% were hospitalised for falls) [15, 17, 19]. Collectively, this high care needs burden and experiences highlight that people accessing care at home might benefit from better preventive, management, and specialist care. Our findings agree with prior research that identified the low use of these services in people in permanent residential aged care facilities and the general older population [31], highlighting the pervasiveness of service access challenges by older Australians. In addition, the percentage of individuals using the examined services was often slightly lower in HCP levels 3–4 than in HCP levels 1–2, which is unexpected given the established greater clinical care needs in recipients of higher HCP levels [30].

We have identified a slight decrease in regular GP attendances and urgent after-hours (for lower level HCPs recipients) utilisation accompanied by an increase of after-hours and nurse practitioner attendances after HCP access. It is possible that the transition to long term care with HCPs, how HCP and additional services are organised by aged care providers, and potential challenges with health and aged care services integration and communication may contribute to the changes observed [32]. While the increase in after-hours attendances after HCP commencement could be related to individuals’ preferences, or potentially lower gap payments due to after-hours services funding structures, it could also be related to an increase in care needs of older people entering HCP [33]. This greater use of after-hours services has also been reported in individuals in permanent residential aged care [31]. However, communication between primary care and aged care providers could influence some of the changes [34]. For example, in December 2022, in response to announced changes to the home care support model in Australia [35], the Royal Australian College of General Practitioners denounced their lack of involvement and consultation on the program development, which may be reflective of past practices [34]. Inadequate coordination between home care and primary care has also been highlighted in international studies [33]. Therefore, it is likely that a number of factors influence these changes and the outcome of this shift to more out of hours attendances requires further clarification.

We have also identified slight decreases in GP health assessments, GP management plans and associated allied health services after HCP access. It is possible that during the transition to HCP, which involves undergoing a number of care needs assessments, HCP recipients may not seek additional assessments to those undertaken by the aged care providers, such as the GP health assessments or chronic disease management plans. However, given the potential benefits of health assessments, which can identify medical, psychological, social, and functional problems, and connect individuals to further care, and GP management plans to improve the coordination and management of chronic diseases affecting these older people [36], their underutilisation and decrease in use is likely a missed opportunity for a cohort of older people with high care needs, often hospitalised, and no longer able to live alone independently [17, 19]. We do note that it is possible that these services are delivered within general GP attendances and not under the studied services, however it is unlikely that the differences in capture of these services would change before and after entry into HCP care, and therefore not explaining the changes after entering care. Additionally, allied health services and medication reviews, known for its significant role in maintaining older individuals’ independency [37], were underutilised and further decreased slightly after HCP access. It is likely that some individuals might have accessed these services through private insurance or through the aged care providers [38], however, further research is required to investigate whether the barriers to allied health access, as highlighted by the Royal Commission into Aged Care Quality and Safety [16], are more pronounced among HCP recipients.

Our study finds that pain and geriatric service utilisation decreased very slightly for HCP recipients while palliative care service access increased slightly after HCP access. These small changes are likely related to the reasons why individuals have sought HCP and what occurred leading up to accessing services- including challenges with function, cognitive impairment, or major health events. While no major changes were identified after HCP entry, the low access in MBS subsidised specialists services before and after HCP is an important finding giving the rising number of HCP recipients and their high risk of poor health outcomes [17, 19]. While we acknowledge that GPs can provide complex health care to older individuals during regular attendances and a number of states and regions offer outpatient geriatric or specialist pain services, how much these services fill the gap is unknown. The documented high risk of hospitalisations in this cohort suggests that these are not addressing all of their geriatric, pain, and palliative care needs [39]. Several challenges associated with specialist service access in Australia have been previously reported, including individuals’ perception of care access [40], availability of the adequate workforce, and costs, which likely contribute to our observations [41].

This study is novel and covers an increasingly high priority cohort of people, which has increased almost 5-fold in 4 years. Our study limitations include the scope of health care services examined, which was limited to those subsidised by the Australian Government, and excluded services accessed privately, through public hospital outpatient clinics, community health services, or through aged care or other providers. However, except for allied health and mental health services [42, 43], which may be accessed more frequently privately, and palliative care, which is more often accessed through hospitals [44], it is unlikely that the other services examined were commonly and consistently accessed through these other mechanisms. We have also interpreted service utilisation as low generally on the basis that individuals accessing HCPs have a high burden of health conditions, multimorbidity, dementia, and recorded challenges with living at home unassisted, who could benefit from some of the services studied. The cohort excluded DVA card holders, which represent 3.8% of the HCP recipients nationally, because they are eligible for a broader range of subsidised health and home support through their entitlements and therefore, their eligibility and access to service(s) is different from other aged care users. We also did not examine service utilisation by Aboriginal and Torres Strait Islanders, who in 2019 were less than 4% of the cohort in HCP, as this requires specific indigenous leadership, governance, and ethics approvals which were not in place for this study. We examined only the most frequently used MBS subsidised GP attendances and acknowledge that some attendances, such as telehealth consultations, which were not widely used during the study period but are increasingly common after COVID-19 [45], were not included. Our study considered only individuals who accessed HCP between 2017 and 2019 due to data availability, and therefore, we are unable to examine more recent service utilisation. It is also possible that external factors (e.g., health and aged care policies, workforce capacity) may have affected the service utilisation evaluated during the study period and unmeasured confounders affected the likelihood of services after care entry and were not captured in our analysis.

Our study strengths include the nationwide coverage of individuals with HCP between 2017 and 2019, rendering our findings generalisable to the older population accessing HCP in Australia. We have used a novel cross-sectoral integrated data source that allowed for the first time in Australia the examination of health care services by HCP recipients [24]. Using this data source, we used leveraged multiple datasets to ascertain study covariates, including aged care eligibility assessments performed by trained clinicians, ensuring greater internal validity of our study measurements.

### Conclusions and implications

We have identified an underutilisation and a generally small but downward trend in the use of important primary and specialist care services by older Australians entering HCP services. With an increasing number of HCP recipients and less permanent residents in aged care facilities, the opportunities for improving their overall health and care management should be maximised, this includes better integration and access of health care. Better health care access and care integration for HCP recipients may be the key to reduce hospitalisations and other important adverse events.

### Electronic supplementary material

Below is the link to the electronic supplementary material.

Supplementary Material 1
