# Supplementary material for: Primary, allied health, selected specialists, and mental health service utilisation by home care recipients in Australia before and after accessing the care, 2017–2019
Source: Aging Clin Exp Res. 2024 Mar 29;36(1):83. doi: 10.1007/s40520-024-02731-9 (PMC10980604; doi:10.1007/s40520-024-02731-9)
Supplement: Supplementary file 1 — Supplementary Material 1 [file 40520_2024_2731_MOESM1_ESM.docx]

**Supplementary Files**

**Supplementary Figure 1. Study cohort selection flowchart**

**
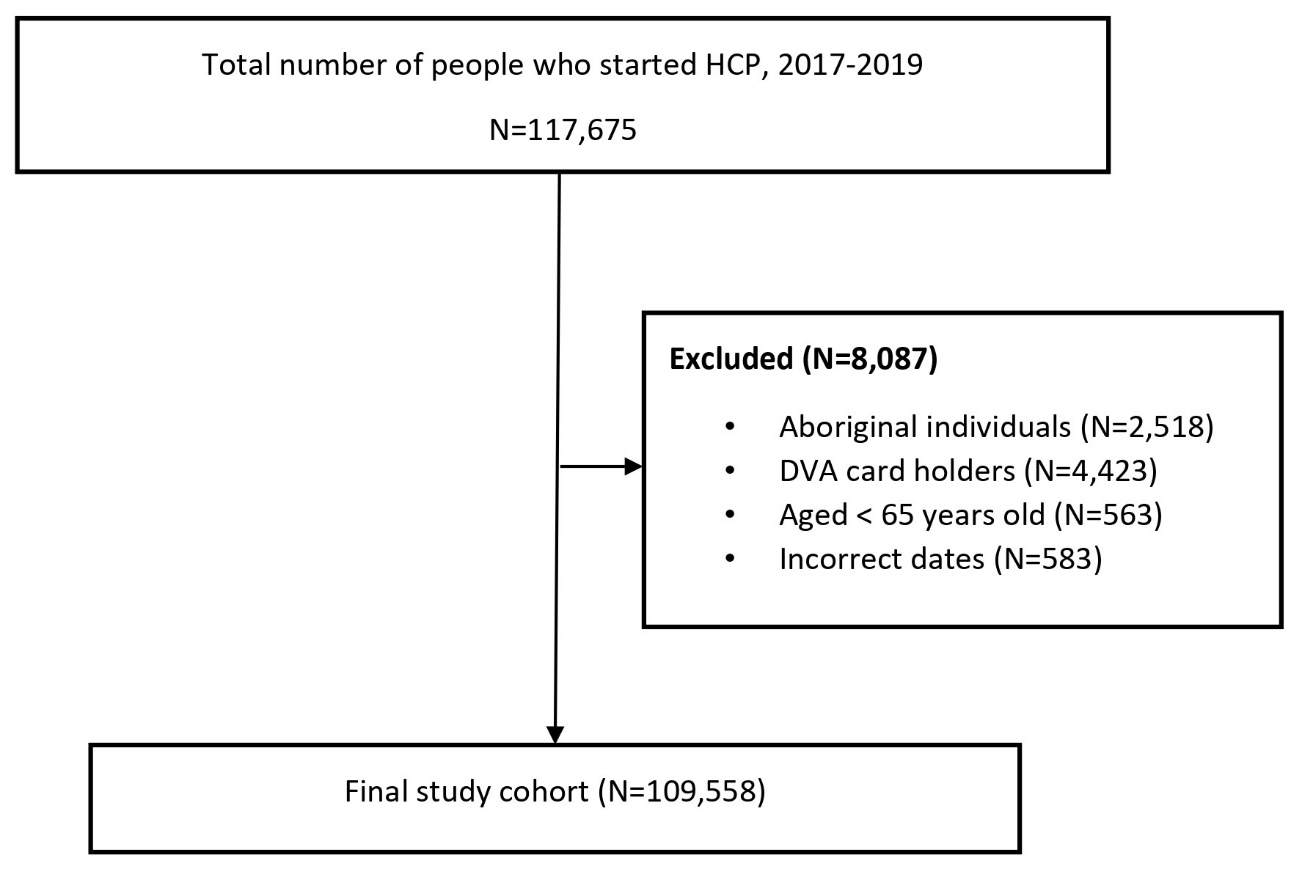
**

**Supplementary Table 1.** Medicare Benefits Schedule group, descriptions, and item codes

| **Health Care Services Group** | **MBS Group** | **MBS Group Descriptions** | **MBS Items** |
| --- | --- | --- | --- |
| General Attendances | A01  A02  A35 | GP attendances  Non-referred attendance to medical practitioner  Medical services at residential aged care facilities | 3, 4, 23, 24, 36, 37, 44, 47  52, 53, 54 ,57, 58, 59, 60, 65  20, 35, 43, 51, 92, 93, 95, 96, 183, 188, 202, 212  90020, 90035, 90043, 90051, 90092, 90093, 90095, 90096, 90183, 90188, 90202, 90212, 90001, 90002 |
|  | A22  A23 | GP after-hours attendance  Non-referred after-hours attendance with medical practitioners | 5000, 5003, 5010, 5020, 5023, 5028, 5040, 5043, 5049, 5060, 5063, 5067 5200, 5203, 5207, 5208, 5220, 5223, 5227, 5228, 5260, 5263, 5265, 5267 |
|  | A11 | Urgent GP attendance after-hours | 585, 588, 591, 594, 597, 598, 599, 600 |
|  | M14 | Nurse practitioners | 82200, 82205, 82210, 82215, 82220, 82221, 82222, 82223, 82224, 82225 |
| Health Assessments  Management Plans | A14 | GP health assessments | 701, 703, 705, 707 |
|  | A15 | GP management plan attendances/team care arrangements and multidisciplinary care plans | 721, 723, 729, 731, 732, 735, 739, 743, 747, 750, 758, 820, 822, 823, 825, 826, 828, 830, 832, 834, 835, 837, 838, 855, 857, 858, 861, 864, 866, 871, 872, 880 |
| Geriatric Medicine Attendances | A28 | Geriatric medicine attendances | 141, 143, 145, 147, 149 |
| Pain and Palliative Medicine | A24 | Pain medicine attendances | 2799, 2801, 2806, 2814, 2820, 2824, 2832, 2840, 2946, 2949, 2954, 2958, 2972, 2974, 2978, 2984, 2988, 2996, 3000, 3003 |
|  |  | Palliative medicine attendances | 3005, 3010, 3014, 3015, 3018, 3023, 3032, 3040, 3044, 3051, 3055, 3062, 3069, 3074, 3078, 3083, 3088, 3093 |
| Allied Health Services | A10 | Optometrical services | 10900, 10905, 10907, 10910, 10911, 10912, 10913, 10914, 10915, 10916, 10918, 10921, 10922, 10923, 10924, 10925, 10926, 10927, 10928, 10929, 10930, 19031, 10932, 10933, 10940, 10941, 10942, 10943, 10944, 10945, 10946, 10947, 10948 |
|  |  |  |  |
|  | A17 | Domiciliary and residential medication management reviews | 245, 249, 900, 903 |
|  | O01 | Dentistry | 51700, 51703, 54001, 54002, 54003, 54004 |
|  | M03 | Allied health component of chronic disease management plan | 10950, 10951, 10952, 10953, 10954, 10956, 10958, 10960, 10962, 10964, 10966, 10968, 10970 |
| Mental Health Services | A08 | Consultant psychiatrist attendances | 288, 289, 291, 293, 296, 297, 299, 300, 302, 304, 306, 308, 310, 312, 314, 316, 318, 319, 320, 322, 324, 326, 328, 330, 332, 334, 336, 338, 342, 344, 346, 348, 350, 352, 353, 355, 356, 357, 358, 359, 361, 364, 366, 367, 369, 370 |
|  | M06 | Psychological therapy services | 80000, 80001, 80005, 80010, 80011, 80015, 80020, 80021 |
|  | M07 | Focussed psychological strategies | 80100, 80101, 80105, 80110, 80111, 80115, 80120, 80121, 80125, 80126, 80130, 80135, 80136, 80140, 80145,80146, 80150, 80151, 80155, 80160, 80161, 80165, 80170, 80171 |
|  | A20 | GP mental health treatment | 2700, 2701,2702, 2712, 2713, 2715, 2717, 2719, 2721, 2723, 2725, 2727 |

Abbreviations: MBS, Medicare Benefits Schedule; GP, General Practitioner; PIP, Practice Incentive Program.

**Supplementary Table 2.** Counts and proportions of Medicare Benefits Schedule subsidised primary care, allied health, mental health, geriatric, pain and palliative services among Home Care Package (HCP) recipients 12 months before and after HCP access

| **Time Interval** | **12 months before** | | | **12 months after** | | |
| --- | --- | --- | --- | --- | --- | --- |
| Service Group / HCP Level | **Total** | **HCP levels 1-2**  **(n=81,500)** | **HCP levels 3-4 (n=28,058)** | **Total** | **HCP levels 1-2**  **(n=81,500)** | **HCP levels 3-4**  **(n=28,058)** |
| **Primary Care** | n (%) | n (%) | n (%) | n (%) | n (%) | n (%) |
| **General Attendances** |  |  |  |  |  |  |
| GP/Medical practitioner attendances | 108,406 (99.0) | 80,742 (99.1) | 27,667 (98.6) | 106,030 (96.8) | 79,188 (97.2) | 26,842 (95.7) |
| Urgent GP attendance after-hours | 10,821 (9.88) | 7,169 (8.80) | 3,652 (13.0) | 9,876 (9.01) | 6,547 (8.03) | 3,326 (11.9) |
| GP/Medical practitioner after-hours attendances | 22,870 (20.9) | 16,272 (20.0) | 6,598 (23.5) | 24,370 (22.2) | 17,396 (21.3) | 6,974 (24.9) |
| Nurse practitioners | 1,507 (1.38) | 1,041 (1.28) | 466 (1.66) | 1,966 (1.79) | 1,382 (1.70) | 584 (2.08) |
| **Health Assessments / Management Plans** |  |  |  |  |  |  |
| GP Health assessments^a^ | 21,284 (29.8) | 16,436 (30.8) | 4,848 (27.1) | 17,854 (24.7) | 13,951 (25.8) | 3,903 (21.5) |
| GP Management plans | 73,139 (66.8) | 55,078 (67.6) | 18,061 (64.4) | 63,116 (57.6) | 48,377 (59.4) | 14,739 (52.5) |
| GP Attendance associated with PIP/Non-referred attendance associated with PIP | 7,182 (6.56) | 5,556 (6.82) | 1,626 (5.80) | 4,877 (4.45) | 3,782 (4.64) | 1,095 (3.90) |
| **Allied Health Services** |  |  |  |  |  |  |
| Optometrical services | 53,242 (48.6) | 41,526 (51.0) | 11,716 (41.8) | 43,684 (39.9) | 34,682 (42.6) | 9,005 (32.1) |
| Comprehensive medication review | 4,896 (4.5) | 3,682 (4.6) | 1,214 (4.4) | 4,285 (4.0) | 3,255 (4.1) | 1,030 (3.7) |
| Dentistry | 290 (0.26) | 218 (0.27) | 72 (0.26) | 2426 (0.21) | 171 (0.21) | 55 (0.20) |
| Allied health service part of CDMP | 57,560 (52.5) | 43,975 (54.0) | 13,585 (48.)4 | 52,002 (47.5) | 40,611 (49.8) | 11,391 (40.6) |
| Podiatry | 47,783 (43.6) | 36,240 (44.5) | 11,543 (41.1) | 44,433 (40.6) | 34,513 (42.3) | 9,920 (35.4) |
| Physiotherapy | 10,600 (9.68) | 8,428 (10.3) | 2,172 (7.74) | 8,016 (7.32) | 6,565 (8.06) | 1,451 (5.17) |
| Dietetics services | 1,566 (1.43) | 1,221 (1.50) | 345 (1.23) | 1,212 (1.11) | 943 (1.16) | 269 (0.96) |
| Exercise physiology | 1,482 (1.35) | 1,134 (1.39) | 348 (1.24) | 1,080 (0.99) | 855 (1.05) | 225 (0.80) |
| Chiropractic | 1,264 (1.15) | 1,056 (1.3) | 208 (0.74) | 981 (0.90) | 831 (1.02) | 150 (0.53) |
| Osteopathy | 587 (0.54) | 490 (0.60) | 97 (0.35) | 489 (0.45) | 401 (0.49) | 88 (0.31) |
| Occupational therapy | 367 (0.33) | 253 (0.31) | 114 (0.41) | 218 (0.20) | 168 (0.21) | 50 (0.18) |
| Speech pathology | 138 (0.13) | 81 (0.10) | 57 (0.20) | 88 (0.08) | 61 (0.07) | 27 (0.10) |
| Mental health services | 15 (0.01) | #^b^ | #^b^ | 11 (0.01) | #^b^ | #^b^ |
| Psychology | 21 (0.02) | 12 (0.01) | 9 (0.03) | 22 (0.02) | 14 (0.02) | 8 (0.03) |
| Audiology | 12 (0.01) | #^b^ | #^b^ | 15 (0.01) | #^b^ | #^b^ |
| **Mental Health Services** |  |  |  |  |  |  |
| GP Mental health treatment | 8,412 (7.68) | 6,492 (7.97) | 1,920 (6.84) | 6,390 (5.83) | 4,999 (6.13) | 1,391 (4.96) |
| Consultant psychiatrist attendances | 3,410 (3.11) | 2,365 (2.90) | 1,045 (3.72) | 2,795 (2.55) | 1,977 (2.43) | 818 (2.92) |
| Focused psychological strategies | 2,255 (2.06) | 1,753 (2.15) | 502 (1.79) | 1,814 (1.66) | 1,435 (1.76) | 379 (1.35) |
| Psychological therapy services | 1,248 (1.14) | 962 (1.18) | 286 (1.01) | 985 (0.90) | 750 (0.92) | 235 (0.84) |
| **Specialist Geriatric, Pain and Palliative Services** |  |  |  |  |  |  |
| Pain medicine | 1,351 (1.23) | 981 (1.20) | 370 (1.32) | 1,061 (0.97) | 775 (0.95) | 286 (1.02) |
| Palliative medicine | 822 (0.75) | 506 (0.62) | 316 (1.13) | 1,486 (1.36) | 969 (1.19) | 517 (1.84) |
| Geriatric medicine | 7,490 (6.84) | 5,375 (6.60) | 2,115 (7.54) | 6,238 (5.69) | 4,590 (5.63) | 1,648 (5.87) |

^a^Only individuals 75+ years old were eligible and only once/year, therefore cohort restricted to 75+ only (12 months before, N=71,324; 12 months after, N=72,230).

^b^Indicates small cell counts (N≤5) that cannot be shown.

Abbreviations: HCP, Home Care Package; GP, General Practitioner; PIP, Practice Incentive Program; CDMP, Chronic Disease Management Plan.
